# Is There a Trojan Horse to Aggressive Pancreatic Cancer Biology? A Review of the Trypsin-PAR2 Axis to Proliferation, Early Invasion, and Metastasis

**DOI:** 10.1089/pancan.2019.0014

**Published:** 2020-02-06

**Authors:** Kjetil Søreide, Marcus Roalsø, Jan Rune Aunan

**Affiliations:** ^1^ Gastrointestinal Translational Research Unit, Laboratory for Molecular Medicine, Stavanger University Hospital, Stavanger, Norway.; ^2^ Department of Gastrointestinal Surgery, HPB Unit, Stavanger University Hospital, Stavanger, Norway.; ^3^ Department of Clinical Medicine, University of Bergen, Bergen, Norway.; ^4^ Faculty of Health and Medicine, University of Stavanger, Stavanger, Norway.

**Keywords:** cancer biology, carcinogenesis, invasion, proliferation, trypsin

## Abstract

**Purpose:** Pancreatic cancer is one of the most lethal of solid tumors and is associated with aggressive cancer biology. The purpose is to review the role of trypsin and effect on molecular and cellular processes potentially explaining the aggressive biology in pancreatic cancer.

**Methods:** A narrative literature review of studies investigating trypsin and its effect on protease systems in cancer, with special reference to pancreatic cancer biology.

**Results:** Proteases, such as trypsin, provides a significant advantage to developing tumors through the ability to remodel the extracellular matrix, promote cell invasion and migration, and facilitate angiogenesis. Trypsin is a digestive enzyme produced by the exocrine pancreas that is also related to mechanisms of proliferation, invasion and metastasis. Several of these mechanisms may be co-regulated or influenced by activation of proteinase-activated receptor 2 (PAR-2). The current role in pancreatic cancer is not clear but emerging data suggest several potential mechanisms. Trypsin may act as a Trojan horse in the pancreatic gland, facilitating several molecular pathways from the onset, which leads to rapid progression of the disease. Pancreatic cancer cell lines containing PAR-2 proliferate upon exposure to trypsin, whereas cancer cell lines not containing PAR-2 fail to proliferate upon trypsin expression. Several mechanisms of action include a proinflammatory environment, signals inducing proliferation and migration, and direct and indirect evidence for mechanisms promoting invasion and metastasis. Novel techniques (such as organoid models) and increased understanding of mechanisms (such as the microbiome) may yield improved understanding into the role of trypsin in pancreatic carcinogenesis.

**Conclusion:** Trypsin is naturally present in the pancreatic gland and may experience pathological activation intracellularly and in the neoplastic environment, which speeds up molecular mechanisms of proliferation, invasion, and metastasis. Further investigation of these processes will provide important insights into how pancreatic cancer evolves, and suggest new ways for treatment.

## Introduction

Pancreatic cancer is one of the most lethal solid organ tumors and is estimated to become one of the top two leading causes to cancer deaths within the next decade.^1^ Patients with pancreatic cancer experience a very poor survival with <5% to 6% being alive >5 years after diagnosis. The lack of real progress in pancreatic cancer over the past decades is of concern, although progress in the characterization at a genomic level has brought the understanding of disease characteristics and heterogeneity forward.^2^ However, pancreatic cancer demonstrates features of aggressive biology; early invasiveness even in smaller tumors with frequent perineural infiltration and a strong desmoplastic stromal response, rendering tumors resistant to most forms of therapy.^3,4^ In a large population-based study of patients with pancreatic cancer, investigators identified a subgroup of patients with small cancers (<0.5 cm) at diagnosis, of which almost one third (31%) already had metastasis at the time of diagnosis.^5^ The propensity to metastasize into the liver is believed to start early and likely involve the tumor stroma, with exosomes or vesicles secreted into the circulation, creating docks for circulating tumor cells to settle into so-called premetastatic niches in the liver.^6^ However, many of these aggressive features are not unique to pancreatic cancer and may only partly explain the uniquely aggressive biology seen in pancreatic ductal adenocarcinoma (PDAC) compared to other solid organ cancers.^7^ Thus, understanding the peritumoral stroma and its interaction with tumor cells and stromal cells is increasingly perceived as an essential clue to defeat this deadly disease.

Trypsin, a digestive enzyme secreted by the pancreas, has been proposed to have a role in cancer growth.^8^ In pancreatic cancer, trypsin may act as a culprit in the development and progression through yet not fully elucidated mechanisms, hypothesized to be an endogenous signal within the pancreatic environment that fosters invasiveness and metastatic potential. Potential mechanisms are discussed, including findings from extrapancreatic cancers and in pancreatic precursor lesions, with the potential for improved understanding of pancreatic cancer.

## An Enemy Within: A Trojan Horse to Cancer Invasiveness?

**FIG. 1.**
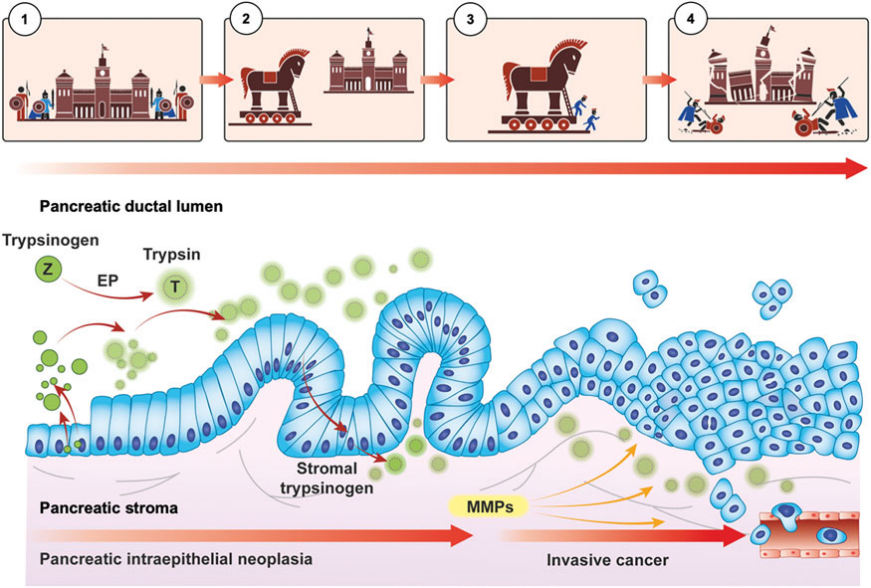
An analogy of trypsin as a Trojan horse in pancreatic cancer. Depicted is to the left (1) the normal checks-and-balances between intraluminal release of trypsinogen only activated into trypsin by EP in the duodenum. This is the analogy to the “siege” or, “closed doors” were indolent/inactive warriors are barred from action due to the active barriers and regulators. With neoplastic transformation (2), trypsin may be prematurely activated either within the cell (which activate cascades), at the luminal side of epithelial cells, or in the stroma due to leaks as integrity of cellular structures weakens (e.g., due to inflammation) or active transport by endovesical transport mechanisms. Trypsin may (3) act as a catalyst to several downstream effects, likely with PAR-2 as an important activator both at the luminal side and in the stroma, through which (4) proliferation, invasion, and metastasis is then facilitated through several molecular mechanisms. The analogy serves to illustrate for educational purposes the likelihood of an activator (enemy) within the cell (city walls) that break down the natural barriers. See main body of text for further details. EP, enteropeptidase; PAR-2, proteinase-activated receptor 2.

In Greek mythology, the story of the “Trojan horse” tells how the Greeks entered Troy and won the war after many years of futile siege. The Greeks did this by building a large wooden horse—containing a force of Greek warriors inside—that was left as a gift to the Trojans, while pretending to leave the city. When taken inside the city, the warriors embarked from the horse and let the rest of the Greek army inside the city walls (Fig. 1).

Metaphorically speaking, a “Trojan Horse” has come to mean any trick or stratagem that causes a target to invite a foe into a securely protected bastion or place, or; refers to subversion introduced from the outside; or, more recently, applied to deceptively benign computer codes that seem like legitimate applications but are written to damage or disrupt a computer program.

Trypsin, it seems, may have the above capabilities to circumvent a siege to a full-blown attack in pancreatic cancer. Intrinsically, trypsin is located within the pancreatic gland by production in the pancreatic acini. Its legitimate location within the cell is under normal circumstances held in a “checks-and-balances” state to avoid premature activation or, activation in the wrong location. However, during neoplastic development, cellular stress and inflammation may lead to release of trypsin into compartments otherwise protected from this enzyme. The premature activation or, even intracellular activation, of other types of proteases has been demonstrated to be important mechanism to disease states through mechanisms hitherto poorly understood or scarcely investigated.^9^ Premature release and activation in the cell or leaks of trypsin into the stroma may facilitate activation of enzyme cascades, leading to cellular proliferation, migration, invasion, angiogenesis, and metastasis (Fig. 1). Such mechanisms have been demonstrated in pancreatitis.^10–13^ Indeed, investigators have identified the activation of trypsinogen within endocytic vacuoles, cellular organelles that appear in pancreatic acinar cells and treated with the inducers of acute pancreatitis.^14^ Endocytic vacuoles are formed as a result of aberrant compound exocytosis and subsequent internalization of postexocytic structures. Notably, endocytic vacuoles can undergo intracellular rupture and fusion with the plasma membrane, providing trypsin with access to cytoplasmic and extracellular targets.^14^ Thus, trypsin may help explain why pancreas cancer is among the most lethal, aggressive form of human cancers, usually presents at a locally advanced stage at time of diagnosis, with an invasive and metastatic nature unfamiliar to most other solid organ cancers.^15^

## Trypsin Is a Trojan Horse in Pancreas Cancer Invasion

Trypsin (or, its proenzyme/zymogen trypsinogen) is a serine protease and pancreatic digestive enzyme secreted and activated in the duodenum, and commonly known to facilitate digestion by cleaving peptide that make up proteins. Also, trypsin has been known for years to be expressed in several isoforms—at least six^16^ of which three are found in humans.^17^ All are found in the pancreas, but some are also found extrapancreatic such as mesotrypsin, which is also expressed in neurological tissues.^18^ Furthermore, trypsin is expressed in cancerous tissues of several organs and has been demonstrated to facilitate proliferation, invasion, and metastasis.^8,19–23^ Notably, trypsin expression in nonpancreas solid organ tumors is related to advanced disease stages and poor prognosis.^8,19,21,24^

One particular characteristic feature of PDAC is the desmoplastic growth pattern and abundant stroma (usually about 70% of the tumor mass) that in part explains the therapeutic resistance and low access to most chemotherapy regimens.^3^ This desmoplastic stroma and hypovascular environment are also increasingly viewed as both a cause to cancer progression and a potential therapeutical target.^25^ Also, the infiltrative nature means that the tumor can include normal pancreas containing exocrine epithelium that secretes digestive enzymes, such as trypsin. Furthermore, studies show that trypsin-sensitive cancer-cell subpopulation express higher levels of slug, snail, vimentin, and N-cadherin while they show a lack of expression of E-cadherin and claudin, being profile characteristic of the epithelial-to-mesenchymal transition.^26^ Epithelial-to-mesenchymal transition confers metastatic properties to cancer cells by enhancing mobility, invasion, and resistance to apoptotic signals.^27^ In cellular culture (breast and colorectal cancer cells), experiments have shown that trypsin-sensitive (easily detached cells based on short wash-out with low concentration trypsin) show cancer stem cell-like features, increased proliferation, and higher self-renewal ability.^26^ Thus, the role of trypsin in pancreatic cancer proliferation, invasion, and metastasis is of clinical relevance. Indeed, it may be that the activation of trypsin in and around stroma and neoplastic cells fosters a more rapid and aggressive biological behavior that is unparalleled to most other solid cancers. The presence of trypsin in this environment may facilitate activation of cell signaling mechanisms that speed the progression and aggressiveness of the tumor.

## Trypsin May Be Involved in the Early Siege and (Pre)neoplasia Development

Trypsin acts through a controlled “checks-and-balances” system,^28^ for which the proenzyme trypsinogen is usually first activated by an enteropeptidase to trypsin (Fig. 1) when entering the duodenum (indeed, a far distance away from the pancreatic acini and endothelial cells), thus preventing premature activation and autodigestion within the pancreatic duct (which may lead to acute pancreatitis). Notably, trypsin is highly elevated in serum of patients with acute pancreatitis,^29^ suggesting leak into or uptake into the blood stream, likely via the capillary system within the gland. Trypsin inhibitors are in place to avoid premature activation in the wrong location. One such inhibitor is pancreatic secretory trypsin inhibitor (PSTI), also called tumor-associated trypsin inhibitor or SPINK1 when investigated outside the pancreas.^30^

Studies have shown somatic mutations of the cationic trypsinogen gene (PRSS1) in patients with (hereditary) chronic pancreatitis and pancreatic cancer.^31^ Although the risk for pancreatic cancer may be lower than previously thought,^31^ it may serve as a model for the role of pathological intracellular activation of trypsin that also play a role in pancreatic cancerogenesis.^32–34^ However, direct mutation in the gene is unlikely to be the explanation in the vast majority of patients with PDAC, although being a likely key component in the risk of families with hereditary pancreatitis.^31,35^

### Trypsin in pancreatic precursor and other neoplastic lesions of the pancreas

Trypsin has proliferative capacity, as demonstrated in several studies.^8,20,21,23^ Thus, trypsin may be involved in early neoplasia development. This is indicated by findings in preneoplastic lesions of the pancreas (intraductal papillary mucinous neoplasia [IPMNs]), where expression of several proteins in pancreatic juice from patients with IPMNs was significantly higher compared with that in other pancreatic diseases. Notably, the most significant protein was PSTI.^36^ Although this study found the increased pancreatic juice expression to be related to IPMNs only (and not pancreatic ductal carcinoma) and the expression of trypsin *per se* was not investigated, it would suggest that the inhibitor is increased in either a response to increased trypsin activity or acts as an upregulated protein by itself and, thus, acts as a pro-neoplastic factor. The fact that upregulation of PSTI may be an effect of increased trypsin expression and/or activation is indirectly confirmed in another study, which found moderate to strong expression of trypsin in preneoplastic lesion of the pancreas, including mucinous cysts and IPMNs.^37^ In a further matched case–control study of over 33,000 inhabitants among which 84 developed pancreatic cancer, there appeared to be a relation between lower levels of PSTI in relation to trypsin for those who developed cancer, suggesting that a loss of inhibitor-function may pose a risk for subsequent pancreatic cancer development.^38^

A further indirect evidence that trypsin may have a role in preneoplasia development and aggressive phenotype comes from studies of a rare type of tumor called intraductal tubulopapillary neoplasm of the pancreas (ITNP). This rare tumor has an overall very good prognosis (unlike PDAC) with most patients being cured by resection, or showing very good long-term outcome, even in presence of invasive components. Notably, this type of tumor in the pancreas stains all negative for trypsin on immunohistochemistry,^39,40^ indicating that the lack of trypsin activation or tissue leak confers a good biology in this instance. Hence, there appears to be a pattern of trypsin expression related to biology of PDAC, related to precursors as in IPMN, but absent in “good” biology tumors such as the ITNPs.

Among the several molecular classes that are suggested in pancreatic cancer^41^ is a squamous group, frequently found in body and tail cancers with poor prognosis.^42^ Currently it is not known whether the squamous phenotype is a distinct group of tumors or represent a continuum in development (e.g., morphological transformation along a continuum). Notably, trypsin has been reported to have a particular role in squamous cancers, where trypsin-2 activated matrix metalloproteinases (MMPs) such as MMP-9 facilitate invasion and metastasis.^43^ While the trypsin-activation of MMPs remains to be specifically investigated in pancreatic cancer, the principle is demonstrated for squamous oral cancer.^44^ However, a recent study found matric metalloproteinases (MMPs 9 and 1) to be among 10 essential so-called hub genes in predicting prognosis in pancreatic cancer.^45^ Also, expression of several MMPs (MMP-1, -2, -7, and -9) were investigated in a set of IPMNs (known precursors to PDAC) and the MMP expression correlated with the histological grade, type, and invasion of these IPMNs, with higher expression score of MMPs conferring a poorer prognosis.^46^

## Proteinase-Activated Receptor 2 as an Important Co-Player

Proteases can also communicate directly to cells by activation of a unique group of transmembrane G-protein-coupled receptors known as protease-activated receptors (PAR). There are four mammalian PARs (PAR-1, -2, -3, and -4), but most interesting in the current setting is PAR-2. PAR-2 is overexpressed in several advanced stage tumors and is activated by trypsin-like serine proteases that are highly expressed or otherwise dysregulated in several types of cancers.^47^ The several mechanisms of action that are found in PAR-2 related cancerogenesis and for several tumors are reviewed in detail elsewhere.^47^

**FIG. 2.**
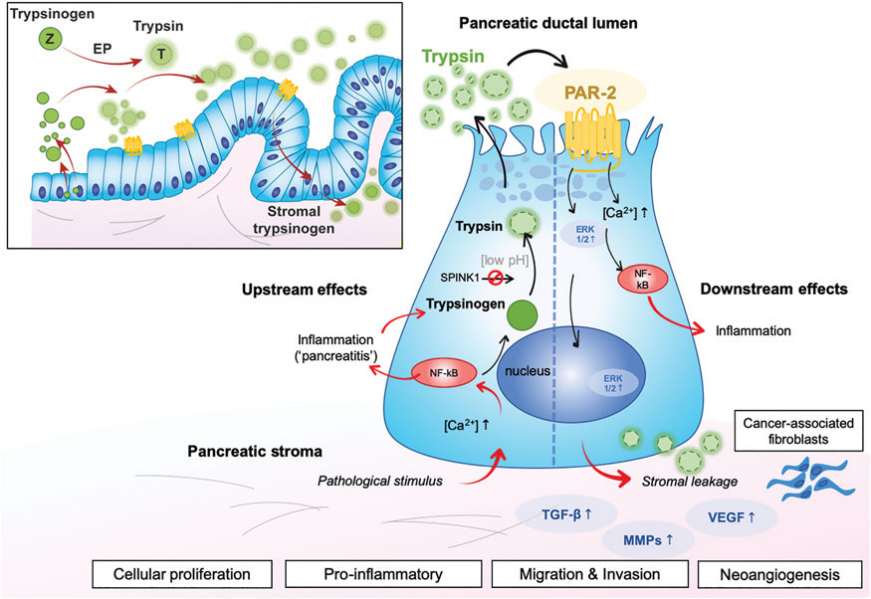
Trypsin activation of PAR-2 with downstream effects. The insert shows how PAR-2 may play a role through early stages of neoplasia progression (PanIN sequences), whereas the upstream and downstream mechanisms are shown in relation to a tentative endothelial cell. Upstream effects may be solicited through pathological stimulus (external or intrinsic to the cell) that further lead to inflammation, trypsin activation, and secretion. PAR-2 may become activated on the luminal side, with ensuing downstream effects on calcium-metabolism, inflammation, nuclear signaling, and resultant protumorigenic effects. Stromal components are involved, including proteinases of the extracellular microenvironment and cancer-associated fibroblasts. Simplistic overview for the purpose of clarity.

Previous studies have established a link between trypsin expression and activated signaling systems—such as PAR-2—with effect on cancer growth of pancreatic cancers.^47–51^ Very high expression of PAR-2 has been found in certain tumor cell lines derived from the lung, colon, and pancreas.^52^ Pancreatic cancer cell lines containing PAR-2 proliferate upon exposure to trypsin, whereas the cancer cell line not containing PAR-2 failed to proliferate upon trypsin exposure.^23^ This suggests that the presence of PAR-2 is needed for trypsin to induce proliferation in cancer cells. Another study found no proliferative activity of PAR-2 on pancreatic cells, but a PAR-2 dependent migration of pancreatic cells.^53^ The same positive association with trypsin and PAR-2 expression has been noted in cholangiocarcinoma cells,^54^ but not in hepatocellular carcinoma, suggesting tumor tissue specific ways of action and influence. The above findings may suggest that PAR-2 may be a target for prevention of migration, invasion, and metastasis (Fig. 2) through pathways activated and involved in pancreatic cancer progression.^55^ A further series of studies reported that PAR-2 may enhance tumorigenesis through crosstalk with transforming growth factor-beta (TGF-beta) signaling to promote TGF-beta1-induced cell migration/invasion and invasion-associated gene expression in PDAC cells.^55^ Further experimental studies in cancer cell lines found that PAR-2 activation drives pancreatic cancer cell migration via an EGF-Src-Rac-p38/mitogen-activated protein kinase kinase/EGF1/2 signaling pathway, which is facilitated by extracellular ATP levels.^53^ Also, PAR-2 is shown to introduce inflammatory signaling^56^ by cytokine release and cyclooxygenase expression,^57^ which may enhance the inflammatory milieu. Hence, several modes of action appear possible by PAR-2 activation with downstream effect on cancer biology (Fig. 2). Of note, one study found that trypsin-PAR-2 signaling contributes to pancreatic cancer pain *in vivo*,^48^ a finding that may be clinically relevant as pain is a common clinical feature for patients with pancreatic cancer.

## Why Has Trypsin Yet to Be Investigated as a Major Player Before in Pancreatic Cancer?

Several methodological issues may explain why trypsin is poorly investigated in pancreatic cancer. First, trypsin is present in abundance in pancreatic tissue due to the physiological exocrine function of the gland, thus the typical dichotomization done in research (high vs. low) may not apply, as it is already present in high amounts even in normal tissue. Second, evaluation of trypsin with immunohistochemistry is difficult as it is not a clear-cut stain by use of most antigens, but often patchy and diffuse with hard to quantify cutoffs and no robust scores for quantification.

Further, gene expression may not reveal significant differences between normal and neoplastic tissue as trypsinogen is omnipresent in pancreatic tissue and ductal epithelial cells due to the physiological function of the exocrine role of the pancreas. In 1 study of 65 patients with pancreatic cancer, 8 (12.3%) patients had mutation in the serine protease 1 gene (PRSS1) coding for the cationic trypsinogen (trypsin 1),^33^ with corresponding higher serum levels of trypsin compared to nonmutated cancers and controls. In another series of patients, mutation of PRSS1 did not seem to play a role in terms of pancreatic cancer development.^34^ It may thus be that mutations in the gene is not the essential change affecting pancreatic cancers, but rather the already present and physiological production of trypsin may be what drives cancer progression. Indeed, at a genetic level it is demonstrated that there is little heterogeneity in driver genes in metastatic disease,^58^ thus other alterations may be essential in contributing to invasion and metastasis after cancer has first developed.

For studies involving RNA investigation, the introduction of trypsinization as part of the experimental process results in a complete degradation of RNA regardless of cell line type, differentiation stage, or passage number.^59^ Thus, findings from RNA studies may not rely on high/low dichotomization but rather on trypsin as present, and therefore acting as a modifier or catalyst of other processes that drive further proliferation and invasive process in pancreatic cancer.

Further, the fact that trypsin is a standard reagent in proteomics experiments but is usually not considered in database searches may lead to a number of false-positive findings. As both lysine and arginine residues are cleavage sites of trypsin, the nearly exclusive use of trypsin as the protein digestion enzyme in shotgun proteomic analyses hinders the detection of transcriptomic alterations, such as seen in junction-spanning peptides.^60^ In proteomic sciences, when cellular protein fractions corresponding to cytosol, membrane, nucleus, and cytoskeleton are further prepared for mass-spectrometry, trypsinization of peptides is a standard step in the process.^61^ Thus, for protein expression, trypsin may not be recognized as an entity of its own in the results, due to the laboratory introduced step of trypsinization that is done to digest proteins before proteomic (or, peptide) investigation. Also, trypsinization may lead to over-abundance of peptides that otherwise would be quantitatively different between groups with high or low levels of trypsin, for example, in pancreatic cancers. This may, despite its purpose, lead to multiple false-positive findings from the experiments itself, as recently demonstrated.^62^ One may thus ask whether trypsinization in such experiments potentially mask the findings of peptides or activated constituents that stem from the native or cancer-driven trypsin-related activity.

The same problem holds for tissue cultures, which are often trypsinized to detach cells and allow for isolation of cells in suspensions. Only more recently has such lab artifacts been recognized to cause differences in results, particularly when investigating metabolites.^63^

Further, novel techniques are in their infancy but may yield new ways to investigate and explore cell behavior. Organoids or 3D cell cultures may be one such opportunity.^64^ Cancer cells have historically been examined using 2D culture methods. However, the morphology, cell-to-cell and cell-to-matrix adhesions, and cellular differentiation of cells grown in 2D culture systems might differ from those growing *in vivo*. Recently, a study using a 3D culture showed that cytokeratin 7, trypsin, CA19-9, and E-cadherin were highly expressed in PK-1 cells but not in PANC-1 cells.^65^ Such differences may have gone undetected in the past and may only now be understood through further research by appreciating variance in cell cultures, cell lines, and in specimens from different patients.

Finally, the more recent revolution related to the association of the microbiome^66^ and fungal^67^ involvement in pancreatic precursors^68^ and carcinogenesis^66,68,69^ may open avenues for new understanding of mechanisms hitherto not explored. It is assumed that the microbiome is present long before a cancer is clinically detected in the pancreas. Currently, no direct evidence exists between any bacteria or fungi related to trypsin activation in early carcinogenesis. However, a clinical study in patients who underwent resection and developed postoperative pancreatic fistulae (POPF) found that *Pseudomonas aeruginosa* and *Enterobacter cloacae* isolated from drainage fluid in patients with clinically relevant (grade B/C) POPF could cause trypsinogen activation.^70^ The investigators further found that trypsinogen activation by *P. aeruginosa* and *E. cloacae* were preventable by the use of a serine protease inhibitor *in vitro* and that a protease in the supernatant from *P. aeruginosa*-positive cultures acted as the trypsinogen activator.^70^ Again, this is extrapolation from data from a different setting, but serves to show that certain bacteria have the ability to activate trypsinogen into trypsin and thereby eliciting effects that may have clinical relevance (Figs. 1 and 2).

## Conclusion

Trypsin may act as a Trojan horse in PDAC biology. While some may find this to be a forced analogy, we do not intend it to be taken literally. The analogy, although imperfect, serves to illustrate that factors leading to an accelerated breakdown within cells and in stroma may hold key information to the poorly understood and exceedingly bad biology of pancreatic cancer (Table 1). Clearly, there must be factors in PDAC that render this form of cancer so exceptionally aggressive and new avenues of research and biological understanding must be sought to improve clinical care. We agree that the analogy may be stretched at this time (due to the actual lack of data to support it beyond the indirect evidence and extrapolation from other tumors). However, as argued in this article, the trypsin effect in cancer may be a consequence of “Trojan horse” transport or dislocation (which may be due to endovesical transport or other pathological dislocation) into compartments where trypsin should not be, including premature intraluminal activation or transport and activation into stromal compartments. Currently, data are incomplete and patchy, we have tried to draw up the lines between existing data and the lack of direct evidence. Connection of the dots and the missing links is still needed in this field.

**Table 1.** Current Understanding and Hypotheses to the Role of Trypsin in Pancreatic Cancer

Trypsin is native to the pancreatic gland and hence pathological exposure may occur early in carcinogenesis
Mechanisms of intracellular activation and extracellular vesicle transport of trypsinogen has been demonstrated in pancreatitis and experimental research. The same mechanisms may be available in neoplasia
Mutation in trypsinogen-gen PRSS1 confers risk for pancreatitis and pancreatic cancer
Trypsin leakage causes cellular proliferation in pancreatitis
Trypsin expression is found to be increased in pancreatic precursors, such as IPMNs
Indolent tumors of the pancreas, such as ITNPs, does not stain for trypsin
Several factors that may be activated by trypsin, including PAR-2 and MMPs, are strongly associated with cell proliferation, invasion, and metastatic potential
PAR-2 is a ligand for trypsin, facilitating cellular growth or migration *in vitro* if both are present
PAR-2-signaling is proinflammatory, which may cause trypsin leakage in to the peritumoral stroma
G-protein-coupled receptors, of which trypsin is a ligand, are related to carcinogenesis
Extrapancreatic data
Increased trypsin expression has been found in aggressive tumors of the digestive tract, prostate, lung, oral cavity, and other tumors.
Expression of trypsin has been related to activation of MMPs and mechanisms in the tumor microenvironment conferring aggressive biology and poor outcome
Nonpancreatic cancer cell lines treated with trypsin gain features seen in cancer stem cells

IPMNs, intraductal papillary mucinous neoplasia; ITNPs, intraductal tubulopapillary neoplasm of the pancreas; MMPs, matrix metalloproteinases; PAR-2, proteinase-activated receptor 2.

Trypsin is present in the pancreatic gland and may experience pathological activation in the (pre-)neoplastic environment, which may speed up molecular mechanisms of proliferation, invasion, and metastasis. PAR-2 may be an essential co-lead for several of these processes. Novel and emerging techniques, including the use of organoid cultures and investigation of the microbiome may give further insight into how trypsin may be activated and play a role in pancreatic carcinogenesis, potentially even from an early onset. Further investigation of these processes will provide important insights into how pancreatic cancer evolves, and suggest new ways for detection, prevention, and treatment.

## Abbreviations Used

IPMNs: intraductal papillary mucinous neoplasia
ITNP: intraductal tubulopapillary neoplasm of the pancreas
MMPs: matrix metalloproteinases
PAR-2: proteinase-activated receptor 2
PDAC: pancreatic ductal adenocarcinoma
POPF: postoperative pancreatic fistulae
PSTI: pancreatic secretory trypsin inhibitor
TATI: tumor-associated trypsin inhibitor
TGF-beta: transforming growth factor-beta
